# Immune desert in MMR-deficient tumors predicts poor responsiveness of immune checkpoint inhibition

**DOI:** 10.3389/fimmu.2023.1142862

**Published:** 2023-04-28

**Authors:** Guoxing Zheng, Yingsi Lu, Zheng Yang, Hong Chen, Qian Liang, Qingqing Zhu, Yan Li, Xing Xiao, Zhuzhen He, Yifan Zhu, Bo Li, Leilei Huang, Nan Dong, Shuang Hu, Yihang Pan, Changhua Zhang, Chengming Zhu

**Affiliations:** ^1^ The Seventh Affiliated Hospital, Sun Yat-sen University, Shenzhen, Guangdong, China; ^2^ Guangdong Provincial Key Laboratory of Digestive Cancer Research, Shenzhen, Guangdong, China; ^3^ The Obstetrics, Shenzhen Amcare Maternity Hospital, Shenzhen, Guangdong, China; ^4^ The First Affiliated Hospital, Sun Yat-sen University, Guangzhou, Guangdong, China

**Keywords:** mismatch repair, tumor immune signatures, somatic mutation, immunotherapy responsiveness prediction, tertiary lymphoid structures

## Abstract

**Background:**

Although many efforts have been devoted to identify biomarkers to predict the responsiveness of immune checkpoint inhibitors, including expression of programmed death-ligand 1 (PD-L1) and major histocompatibility complex (MHC) I, microsatellite instability (MSI), mismatch repair (MMR) defect, tumor mutation burden (TMB), tertiary lymphoid structures (TLSs), and several transcriptional signatures, the sensitivity of these indicators remains to be further improved.

**Materials and methods:**

Here, we integrated T-cell spatial distribution and intratumor transcriptional signals in predicting the response to immune checkpoint therapy in MMR-deficient tumors including tumors of Lynch syndrome (LS).

**Results:**

In both cohorts, MMR-deficient tumors displayed personalized tumor immune signatures, including inflamed, immune excluded, and immune desert, which were not only individual-specific but also organ-specific. Furthermore, the immune desert tumor exhibited a more malignant phenotype characterized by low differentiation adenocarcinoma, larger tumor sizes, and higher metastasis rate. Moreover, the tumor immune signatures associated with distinct populations of infiltrating immune cells were comparable to TLSs and more sensitive than transcriptional signature gene expression profiles (GEPs) in immunotherapy prediction. Surprisingly, the tumor immune signatures might arise from the somatic mutations. Notably, patients with MMR deficiency had benefited from the typing of immune signatures and later immune checkpoint inhibition.

**Conclusion:**

Our findings suggest that compared to PD-L1 expression, MMR, TMB, and GEPs, characterization of the tumor immune signatures in MMR-deficient tumors improves the efficiency of predicting the responsiveness of immune checkpoint inhibition.

## Introduction

In the past two decades, immunotherapy of cancer has achieved great advances, reflecting the crucial functions of the immune system against cancer progression. Although immunotherapy, especially through the blockade of immune checkpoints such as programmed death-1 (PD-1) and its ligand programmed death-ligand 1 (PD-L1), has been widely applied in the treatment of a broad range of human cancers, the proportion of responsive patients experiencing immunologic eradication of cancer remains limited (1).

One important challenge is to predict responsiveness before conducting checkpoint blockade immunotherapy. Many efforts have proved the possibility of anticipating responses to cancer immunotherapy (2–4). The expression of PD-L1 emerges as a valuable indicator (5), but PD-L1 alone is insufficient for selecting patients who may benefit from immunotherapy (6). Tumor mutation burden (TMB) displayed significant associations with immune checkpoint inhibitor (ICI) responses in a variety of tumor types but not in particular cancers such as Merkel cell carcinoma and rectal colon cancer (RCC) (7). TMB higher than 37.4 mutations/Mb appeared to have a very high success rate within high microsatellite instability (MSI-H) metastatic colorectal cancer and may be used to stratify patients for the possibility of responding to ICIs (8). However, chemotherapy contributed to the acquisition of hypermutated burden but did not enhance the response to PD-1 blockade in mismatch repair (MMR)-deficient gliomas (9), reflecting the suboptimal choices of TMB and MMR as predictive markers for immunotherapy. Furthermore, another study illustrated that Lynch syndrome (LS) patients exhibited a striking immune activation independent of mutation burden, neoantigen generation, and MMR status (10). These findings suggest that neither TMB nor MMR alone can be reliable indicators of immunotherapy. Other potential markers for the prediction consist of tumor heterogeneity (11), circulating tumor DNA (12), B cells (13), and distinct T-cell subsets (14, 15).

One important step to improve the effectiveness and accuracy of response anticipation is the integration of various features including CD8^+^ T-cell infiltration pattern, expression of PD-1/PD-L1, and assessment of signaling pathways like interferon gamma (IFNγ), transforming growth factor beta (TGFβ), and fatty acid metabolism (16). Accordingly, tumor immune signatures (16) have three subtypes: inflamed tumors, immune excluded tumors, and immune desert tumors, with their distinct features. Inflamed tumors are primarily associated with immune responses like IFNγ signaling, high PD-L1 expression level, the presence of tumor-infiltrating lymphocytes (TILs), B cells, and intact antigen presentation. Immune excluded tumors are defined by their reactive stromal biology, a physical barrier for exclusion of T cells (17), the signature of TGFβ signaling, and tumor angiogenesis. Immune desert tumors are devoid of lymphocyte infiltration and are primarily characterized by increased fatty acid metabolism and neuroendocrine features. The classification of tumor immune signatures has been proven to be effective in predicting the responsiveness of anti-PD-L1 therapy across a range of cancers, especially in non-small cell lung cancer (NSCLC) (1) and metastatic urothelial cancer (mUC) (17), with immune desert tumors being the poorest responders (18).

LS is caused by autosomal dominant heterozygous germline mutations in one of the MMR genes, mostly MLH1, MSH2, MSH6, or PMS2 (19). The defect of the DNA repair system always leads to MSI, increases risks for malignancies, and accelerates neoplastic progression (19, 20). LS is found in a spectrum of cancers, mainly in colorectal cancers and endometrial cancers (19–21). Here, we apply integrated methods to predict the response of immune checkpoint therapy (ICT) in MMR-deficient tumors including LS through immunohistology and signaling pathway analysis of intratumor transcriptome, and find that the tumor immune signatures are featured with distinct immune cell populations and TLS status and strongly correlated with tumor somatic mutations, cancer development, patient survival, and the response to immunotherapy. The findings provide references and guidance for successful immunologic elimination of cancer cells based on immune checkpoint inhibition.

## Materials and methods

### Patients

The colon tumors and adjacent normal tissues from 10 LS patients carrying germline mutations of the MMR genes MLH1 or MSH2, and endometrial tumor and adjacent normal tissues from one of these patients were collected at the Seventh Affiliated Hospital, Sun Yat-sen University. These samples were then analyzed using immunohistochemistry (IHC), whole-genome transcriptomic analysis, and whole exome sequencing (WES). Patient consents were obtained following the guidelines approved by the Medical Ethics Committee of the Seventh Affiliated Hospital of Sun Yat-sen University. Formalin-fixed paraffin-embedded (FFPE) tissue specimens of 82 colorectal cancer patients with MMR deficiency were used for IHC analysis. The characteristics of these patients are provided in **Table S1**.

### RNA-seq

The total RNA of tumors and adjacent normal tissues in patients with LS was isolated with the RNeasy Mini kit (QIAGEN) after grinding of tissues in liquid nitrogen. The quality, integration, and quantity of obtained RNA were assessed *via* Nanodrop (Thermal), Qubit (Thermal), and agar gel running. Following the manufacturer’s manual, qualified RNA was subjected to library construction with TruSeq^®^ RNA LT Sample Prep Kit v2 (Illumina). The cDNA library was ligated to an adaptor and purified with AMPure XP Beads (Beckman). The second-generation sequencing of the obtained library on PE150 was performed using Hiseq3000 (Illumina). The average number of raw bases per specimen was over 6 GB. The percentage of bases having scores > Q30 for single and paired-end reads were higher than 90% in all the data obtained.

### Immunohistology

The tumor and adjacent normal tissues from patients with LS were collected, fixed, and embedded in paraffin. The tumor specimens with MMR protein defects in colorectal patients were fixed and embedded with paraffin. The 8-μm paraffin sections were stained with Abcam antibodies of CD3 (Ref# ab16669), CD8 (Ref# ab4055), PD-1 (Ref# ab52587), PD-L1 (Ref# ab213524), and human leukocyte antigen (HLA) I (Ref# ab23755), respectively, and counterstained with hematoxylin. The antibody-specific staining on the slides was captured with a Leica DM4B microscope system.

### Tumor immune signature analysis

According to the summary by Priti Hegde and Daniel Chen in *Immunity* (16), the tumor immune signatures have three subtypes: inflamed, immune excluded, and immune desert, with their distinct features and the spatial localization of immune cells (CD8- or CD3-positive cells in this study) infiltrating the tumor. The signaling pathways arising from Gene Ontology (GO), Kyoto Encyclopedia of Genes and Genomes (KEGG), and Reactome function analysis of the target gene set were matched with the distinct tumor immune signatures, especially the dominant ones including IFNγ signaling, PD-L1 expression, the prevalence of TILs, B cells, and intact antigen presentation in inflamed tumors; TGFβ signaling and tumor angiogenesis in immune excluded tumors; and fatty acid metabolism and neuroendocrine in immune desert tumors. The immune cell infiltration patterns were also defined with the defect of immune cells in the immune desert, enrichment of immune cells in the surrounding regions of immune excluded tumors, and highly dispersing immune cells in inflamed tumors.

### The combination therapy of patients

One month after the tumors were surgically removed, the imaging assessment showed progressive disease (PD) in the patient. The immune signature has identified the tumor as immune excluded. The patient with colon cancer of LS received six cycles of combination therapy of anti-VEGF, FOLFIRI, and anti-PD-1 at each cycle for 3 weeks, then four cycles of combination therapy of anti-VEGF and anti-PD-1 at each cycle for 4 weeks. Dosage of combination therapy of anti-VEGF, FOLFIRI, and anti-PD-1: anti-PD-1 sintilimab injection [Xinda Biopharmaceutical (Suzhou) Co., Ltd, Approval No. gyzz S20180016] 200 mg intravenous (iv) + anti-VEGF bevacizumab injection [Roche Pharma (Switzerland) Ltd. Avastin, Import Drug Registration Certificate No. S20170035] 200 mg iv + irinotecan hydrochloride injection (Jiangsu Hengrui Pharmaceutical Co., Ltd, Approval No. gyzz H20061276) 130 mg iv + 5-FU first dosage 0.56 g iv and then 3.3 g iv maintained for 46 h. Dosage of combination therapy of anti-VEGF and anti-PD-1: sintilimab injection 200 mg iv + bevacizumab injection 200 mg iv.

### Immunofluorescence staining

The tumor and adjacent normal tissues from patients with LS were collected, fixed, and embedded in paraffin. The 8-μm paraffin sections were stained with TSAPLus fluorescent triple staining kit (Servicebio, Cat# G1236) following the manufacturer’s protocol. Abcam antibodies of CD23 (Ref# ab92495), CD20 (Ref# ab64088), and DAPI were used. The antibody-specific staining on the slides was captured with a Zeiss LSM880 confocal microscope system.

### GO, KEGG, and Reactome function analysis of the target gene set

GO function analysis on the differentially expressed genes (DEGs) or somatic mutation genes was performed based on TopGO software (http://www.bioconductor.org/packages/release/bioc/html/topGO.html), in which the target genes were selected from all the gene lists. The online KEGG pathway database (http://www.genome.jp/kegg-bin) was applied for KEGG pathway functional enrichment analysis of the target gene set or somatic mutation genes. Additionally, the Reactome database that integrates the various reactions and biological pathways in human was utilized for functional analysis of the target gene set or somatic mutation genes. Whether the function set of GO, KEGG, and Reactome was significantly enriched in the target gene list was determined by the *p*-value calculated by Fisher’s exact test. The *p*-value was further corrected by Benjamini and Hochberg multiple tests to control the false discovery rate (FDR). The corrected *p*-values less than 0.05 were annotated significant in all these three function analyses.

### Deconvolution analysis of RNA-seq data

We used the Cell Fractions module of CIBERSORTx to estimate the percentage of the overall immune cells in each sample. The single-cell reference used was the colorectal cancer samples from Qian et al. (“A pan-cancer blueprint of the heterogeneous tumor microenvironment revealed by single-cell profiling”) (22). The types of immune cells annotated in the reference are T cells, B cells, mononuclear phagocytes, plasma cells, and mast cells. The sum of all immune cell percentages estimated by CIBERSORTx for each sample was used as the overall percentage of immune cells in that sample, denoted as a.

The CIBERSORT algorithm (23) is used to infer the relative proportion of 22 infiltrating immune cells from the normalized gene expression data. Briefly, the FPKM normalized gene expression datasets were uploaded to the CIBERSORT website (http://cibersort.stanford.edu/). The algorithm was run in 1,000 permutations using the default feature matrix. CIBERSORT used Monte Carlo sampling to derive a *p*-value for the deconvolution of each sample. The relative percentage of each immune cell type in LM22 was estimated by CIBERSORT for each sample, denoted as b. The absolute percentage of each immune cell type in LM22 of each sample is equal to a * b.

### Whole exome sequencing

The genomic DNA of tumors and adjacent normal tissues in LS patients was prepared with the Axyprep genomic DNA miniprep kit (animal tissues and human tissues) following the manufacturer’s instructions. The quality and quantity of obtained DNA were evaluated *via* Qubit (Thermal) and electrophoresis on agar gel. The genome was subsequently submitted to library construction with the TruSeq^®^ DNA LT Sample Prep Kit v2 (Illumina). Then, the exon enrichment was performed *via* the Nimblegen Exome Kit V4 (Roche). After quantification, the exon library was sequenced on Hiseq3000 (Illumina). The data with Q30 (the proportion of bases with 99.9% accuracy) higher than 80% were accepted and further analyzed in the following procedures.

### Somatic mutation detection

MuTect2 (http://software.broadinstitute.org/cancer/cga/mutect) was utilized for somatic mutation detection. The filter criteria included the following: (1) the sequencing depth of cancer and adjacent tissues is ≥10; (2) the number of reads supporting this variation in tumors is ≥3; (3) the allele frequency of this variation in tumors is ≥0.05; (4) the allele frequency of this mutation in adjacent normal tissues is ≤0.01; and (5) the filter is equal to pass.

### GEPIA of immune genes in the TCGA database

Gene expression profiling interactive analysis (GEPIA, http://gepia.cancer-pku.cn/) was applied to determine the expression of immune genes including CD8, CD3, PD-L1, and PD-1 in colon adenocarcinoma (COAD) and rectum adenocarcinoma (READ). The level of different cancer stages was analyzed. The overall survival of patients was compared in high-expression and low-expression groups of each gene.

### Statistics

Proportions for categorical variables were compared using the chi-square test, and Fisher’s exact test was used when the data were limited. *p* < 0.05 was considered statistically significant.

## Results

### It is necessary to distinguish tumor immune signatures before immune checkpoint therapy

**Figure 1:**
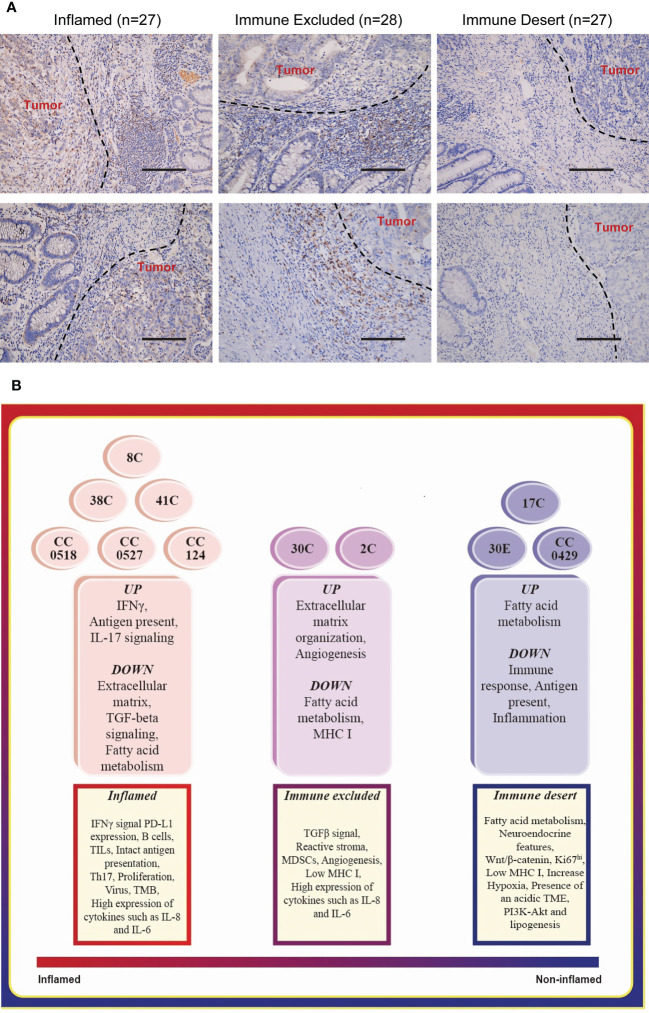
Typing the tumor immune signatures in MMR-deficient tumors with histology and transcriptome profile, respectively. **(A)** The representative images of immunohistology of CD8 staining on MMR-deficient tumors exhibit the patterns of all three tumor immune signatures: immune desert, immune excluded, and inflamed. Magnification: 50×, scale bar: 100 μm. **(B)** Functional enrichment of mRNA expression profile suggests the basic features of the three tumor immune signatures.

The spatial localization of T lymphocytes infiltrating the tumor is a critical basis of categorization of tumor immune signature (16, 24). These were indicated as the following: inflamed tumors, T lymphocytes dispersed in the whole tumor regions; immune excluded tumors, T lymphocytes were rich at the surrounding stroma but excluded from tumor regions; and immune deserts tumors—devoid of T lymphocytes. We collected the FFPE tumor tissues of 82 colorectal cancer patients (**Table S1**), which exhibited the defect of at least one of the four MMR proteins indicated in LS, namely, MLH1, MSH2, MSH6, and PMS2. The spatial localization of infiltration T lymphocytes—one key feature of tumor immune signatures—was distinguished by the staining of CD8, which has been applied in defining tumor immune signatures (1, 17). Among these, 27 specimens were classified as inflamed tumor (**Figure 1A**, **Table S1**), 28 specimens were defined as immune excluded tumors (**Figure 1A**, **Table S1**), and 27 tumors were the immune desert tumors that displayed no or very few CD8 staining (**Figure 1A**, **Table S1**). The proportion of immune desert tumors (27/82), which yielded the worst responses to anti-PD-L1 therapy (1, 16), is quite high among these patients, suggesting the necessity of identifying tumor immune signatures before ICT.

The chi-square test was performed to analyze the correlation between the immune signatures and pathologic features of LS patients (**Table 1**). While no correlation was found in age and gender, the tumors of the immune desert subtype were significantly associated with metastasis (*p* < 0.0001) and large tumor sizes (diameter ≥3 cm, *p* < 0.0001). Notably, the immune excluded and inflamed tumors together markedly correlated with middle and middle high differentiated adenocarcinomas (*p* = 0.014). Together, immune desert tumors are more malignant, with low differentiation, increasing tumor sizes, and higher metastasis ratios. This suggested that the application of tumor immune signatures in predicting the responsiveness of ICT is reasonable.

**Table 1:** The correlation between immune signature and pathologic features in patients with MMR deficiency.

Features	No. of cases	Immune Signature		*p*-value (*χ* ^2^ tests)
		Desert	Excluded + Inflamed	
Total	82	27 (32.9%)	55 (28 (34.2%) + 27 (32.9%))	
Age (years)				
<50	35	12 (14.6%)	23 (9 (11%) + 14 (17.1%))	
≥50	47	15 (18.3%)	32 (19 (23.2%) + 13 (15.9%))	0.821
Gender	26	9 (11%)	17 (10 (12.2%) + 7 (8.5%))	
Female				
Male	56	18 (22%)	38 (18 (22%) + 20 (24.4%))	0.825
Metastasis				
Yes	22	16 (19.5%)	6 (3 (3.7%) + 3 (3.7%))	
No	60	11 (13.4%)	49 (25 (30.5%) + 24 (29.3%))	<0.0001
Tumor size				
<3 cm	73	19 (23.2%)	54 (27 (32.9%) + 27 (32.9%))	
≥3 cm	9	8 (9.8%)	1 (1.2%)	<0.0001
Location1	59	18 (22%)	41 (20 (24.4%) + 21 (25.6%))	
Colon				
Others	23	9 (11%)	14 (8 (9.8%) + 6 (7.3%))	0.455
Location2	10	6 (7.3%)	4 (2 (2.4%) + 2 (2.4%))	
Cecum				
Others	72	21 (25.6%)	51 (26 (31.7%) + 25 (30.5%))	0.073
Type MDA+MHDA	37	7 (8.5%)	30 (19 (23.2%) + 11 (13.4%))	
Others	45	20 (24.4%)	25 (9 (11%) + 16 (19.5%))	0.014
Stage T1+T2	3	0	3 (2 (2.4%) + 1 (1.2%))	
T3+T4	19	6 (7.3%)	13 (6 (7.3%) + 7 (8.5%))	0.833
Infiltration WLIW	49	17 (20.7%)	32 (18 (22%) + 14 (17%))	
Others	33	10 (12.2%)	23 (10 (12.2%) + 13 (15.9%))	0.678

MDA, middle differentiated adenocarcinoma; MHDA, middle high differentiated adenocarcinoma; WLIW, whole layer of intestinal wall.

### Two methods of typing tumor immune signatures from different individuals were consistent

We collected colon tumors and adjacent normal tissues from 10 LS patients (**Table S2**) and endometrial tumor (30E) and adjacent normal tissues from one of these patients. The whole genome RNA-seq of these specimens was carried out. To confirm the applications of tumor immune signature categorization, we performed various functional enrichment analyses of DEGs, including GO, KEGG, and Reactome. Matching the enriched pathways in each tumor (**Figure 1B**, middle) to the features of immune signatures (**Figure 1B**, bottom) characterized six colon tumors, namely, 38C, 8C, 41C, CC0518, CC0527, and CC124, as the inflamed subtype; two colon tumors, 30C (colon tumor of patient #30) and 2C, as immune excluded tumors; and the remaining specimen, 17C, CC0429, and 30E (endometrial tumor of patient #30), as immune desert tumors. The results suggested that the tumors from different individuals or organs of LS have personalized tumor immune signatures.

**Figure 2:**
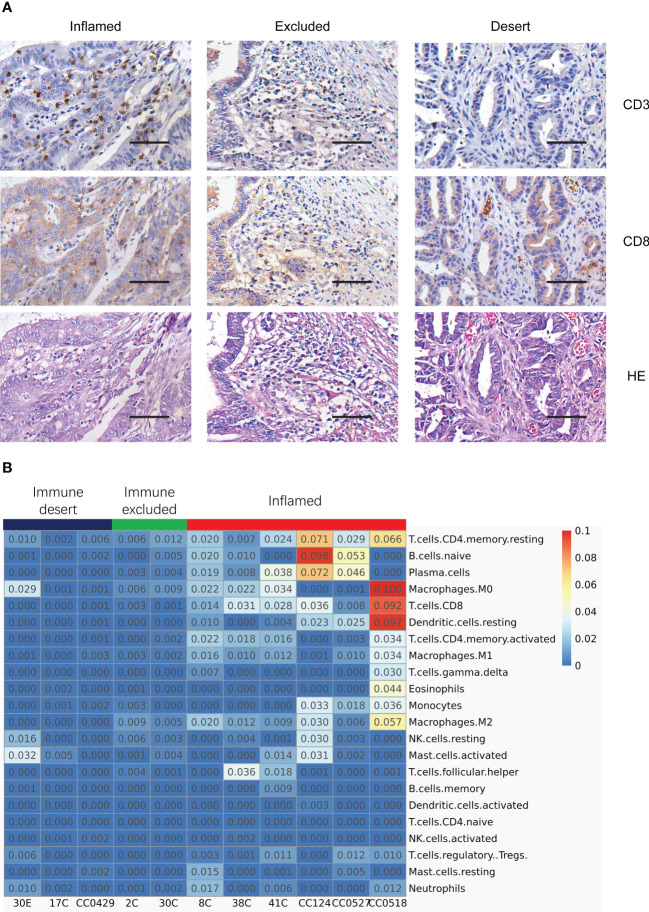
Typing the tumor immune signatures in MMR-deficient tumors by the integration of histology and transcriptome profile. **(A)** Immunostaining of CD3, CD8, and HE exhibits the patterns of various tumor immune signatures in different MMR-deficient tumors. Magnification: 50×, scale bar: 100 μm. **(B)** Deconvolution analysis of RNA-seq data reveals the infiltration of different populations of immune cells in various tumor immune signatures. The relative fraction of 22 immune cell types is inferred by CIBERSORT.

To assess the spatial distribution of T cells in LS specimens, the immunostaining of CD3, CD8, and HE was conducted to label the T lymphocytes in tumors. As expected, the distribution of T cells matched with the tumor immune signatures derived from the assay of functional enrichment of transcriptome. The inflamed tumor was infiltrated by lymphocytes, immune excluded colon tumors associated with the surrounding embedding of immune cells, and the immune desert tumor was devoid of TILs (**Figure 2A**). In addition, the expression of PD-L1 and MHC I is a critical indicator in the classification of tumor immune signature (17); thus, their IHC was also conducted. The spatial distribution of PD-L1, its receptor PD-1, and MHC I (**Figure S1**) was similar to CD3^+^ T lymphocytes. It seems that the spatial distribution of PD-L1 is more critical than the expression level itself in the prediction of ICT responses. Taken together, IHC of CD3, PD-1, PD-L1, and MHC I confirmed the personalized tumor immune signatures in different patients and organs. Additionally, this suggests that the typing of tumor immune signature *via* the intratumor transcriptomic profile is consistent with the typing by the distribution of T lymphocytes.

### The tumors of various immune signatures had different patterns of immune cell infiltration

To further dissect the constitution of immune cell types in the tumor microenvironment (TME), deconvolution analysis of RNA-seq data was performed using the CIBERSORT algorithm. While inflamed tumors were highlighted with the highest percentage of both CD8^+^ T cells and plasma cells and a relatively higher level of activated CD4 T cells, resting dendritic cells (DCs), and naïve B cells (**Figure 2B**), immune excluded tumors had a very weakly higher level of M2 macrophage, CD8^+^ T cells, and plasma cells (**Figure 2B**), and one sample of immune desert tumors had a relatively higher level of activated M0 macrophage, NK cells, neutrophils, and activated mast cells (**Figure 2B**). Actually, the other cell populations, including M2 macrophages, DCs, naïve B cells, NK cells, and mast cells, appeared to be very low across all tumor subtypes. The deconvolution analysis illustrated that the inflamed tumors were associated with the strongest infiltration of various immune cell clusters including T cells and B cells. The results also suggest that the relative immune reactive activities marked by the level of CD8^+^ T cells and plasma cells are highest in inflamed tumors, low in immune excluded tumors, and lowest in immune desert tumors, indicating the potential difference of responsiveness to immune checkpoint inhibition.

### The tumor immune signatures were comparable to tertiary lymphoid structures

**Figure 3:**
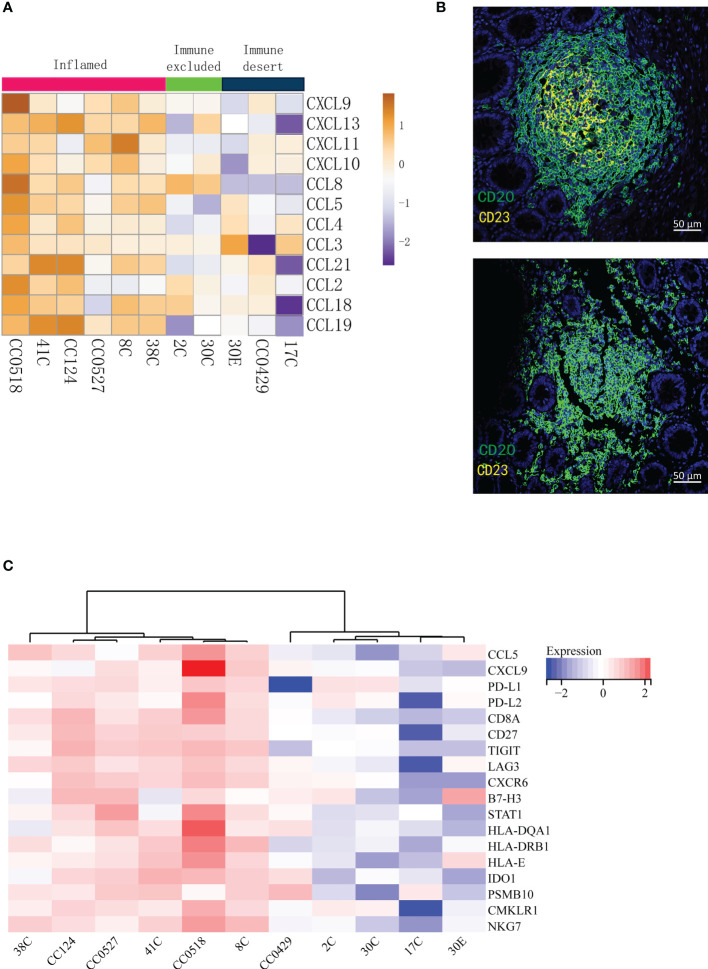
The tumor immune signatures are associated with the infiltration of distinct populations of immune cells and with the status of tertiary lymphoid structures. **(A)** Analysis of the 12-chemokine transcriptional expression suggests that the presence of tertiary lymphoid structures varies in different tumor immune signatures. **(B)** Multiplex IF of CD20 and CD23 shows that while mature TLSs (top) are found in the tumors of immune excluded and inflamed, immature TLSs (bottom) are related to the desert tumors. **(C)** The GEPs cannot distinguish immune excluded and immune desert tumors. The 18-gene T inflamed gene expression profiles (GEPs) are applied to the transcription profiles of 11 tumors; the cluster of GEPs is presented as a heat map.

Tertiary lymphoid structures (TLSs) are ectopic germinal center-like lymphoid organs that develop at sites different to lymphoid tissues like tumors (25). TLSs consist of a T cell-rich zone with follicular DCs juxtaposing a B-cell follicle surrounded by plasma cells. Recently, it has been reported that TLSs can be applied to predict the efficacy of ICI in solid tumors (26–29). In particular, mature TLSs (mTLSs) with CD23^+^ follicular DCs inside are favored by immune checkpoint inhibition, while immature TLSs (iTLSs) without CD23^+^ follicular DCs are not (30). From the deconvolution analysis of RNA-seq data, we noticed that the lymphocytes related with TLSs like T cells, DCs, and plasma cells were enriched in inflamed tumors. Thus, we asked whether the tumor immune signatures were related to TLSs in LS. The 12-chemokine signature (31) in transcriptome for the detection of TLSs in colorectal cancer was applied. As expected, the inflamed tumors in tumor immune signatures were rich in TLSs, indicated by the highest expression of most chemokines (**Figure 3A**). Furthermore, multiplex immunofluorescence staining of the two typical markers of TLSs—CD20 (B cells) and CD23 (DCs)—was performed to validate the presence of TLSs in LS tumors. The mTLSs (**Figure 3B**, top) were exclusively found in the inflamed tumors and immune excluded tumors, while the immune desert tumors only carried iTLSs (**Figure 3B**, bottom). The data revealed that the tumor immune signatures are consistent with the status of TLSs in the tumors of MMR deficiency.

### T cell-inflamed gene expression profiles could not distinguish the difference between immune excluded and immune desert tumors

In 2017, Ayers et al. developed a set of IFN-γ-related mRNA profile to predict the clinical response to PD-1 blockade in melanoma (32). The final 18-gene T cell-inflamed gene expression profiles (GEPs) were more sensitive than PD-L1 IHC to detect responders to anti-PD-1 therapies with an area under the receiver operating characteristic (ROC) curve of 0.75. Although the GEFs and TMB had a low correlation, their joint prediction could be utilized in identifying responders to the PD-1 antibody in pan-tumors (33). Importantly, the GEPs had a strong correlation (*r* > 0.9) with several previously published transcriptional signatures of TME including chemokine signature (31), Immunoscore (34), and cytolytic activity (35). In order to compare the sensitivity of the immune signatures to previously published transcriptional signatures, the analysis of T cell-inflamed GEPs was applied to our patients. The analysis (**Figure 3C**) distinguished the inflamed specimens from non-inflamed specimens very well. However, the differences between immune excluded and immune desert tumors were not obviously determined (**Figure 3C**). Therefore, the tumor immune signatures may offer a more optimal choice than GEPs in identifying the responders to ICT.

### The tumors from different organs of the same patient had unique immune signatures

**Figure 4:**
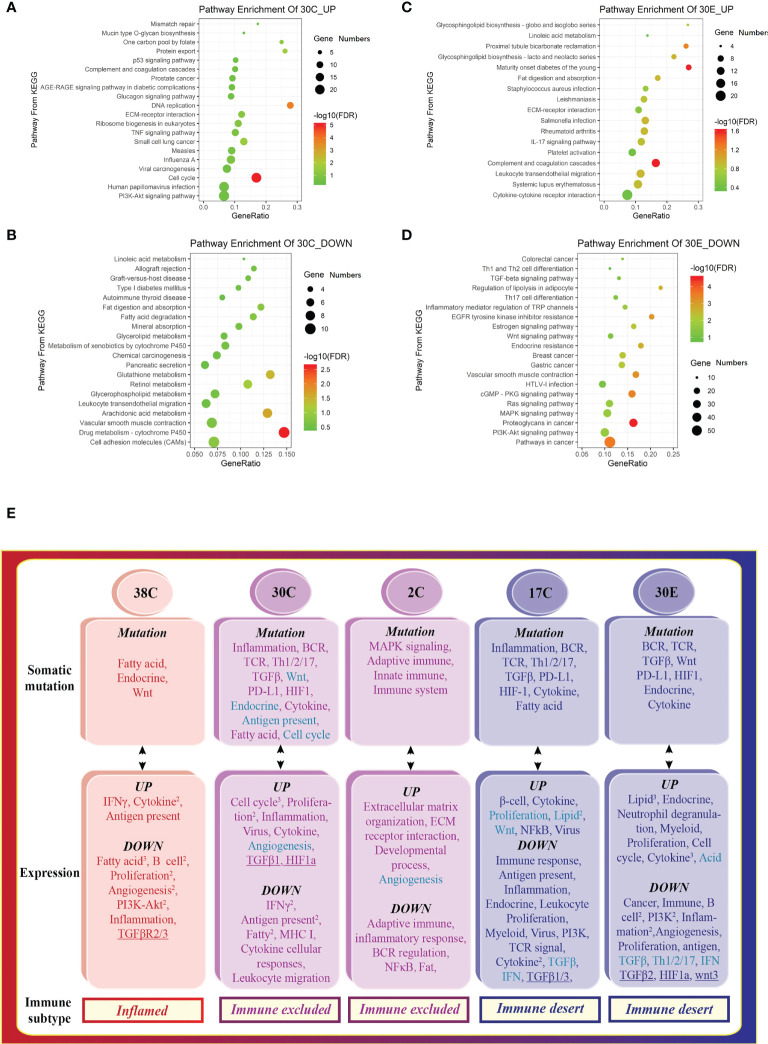
The tumors from different organs of the same patient display distinct tumor immune signatures. Scatterplot of enriched Kyoto Encyclopedia of Genes and Genomes (KEGG) pathways of **(A)** downregulated and **(B)** upregulated DEGs in colon tumors, indicating the activation of the cell cycle, proliferation, and inflammation. Scatterplot of enriched KEGG pathways of **(C)** downregulated and **(D)** upregulated DEGs in the endometrial tumor, suggesting the activation of lipid metabolism. 30E—endometrial tumor of patient #30. The vertical axis represents the KEGG pathway. The horizontal axis represents the percentage of DEGs in the total number of genes involved in certain KEGG pathways. The size of the bubble indicates the number of DEGs enriched in this item, and the color of the dots indicates the range of –log10 (FDR). **(E)** The tumor somatic mutations contribute to the variation of the tumor immune signatures in Lynch syndrome. The mRNA expression patterns that are opposite to somatic mutation profiles in different tumors correspond to various tumor immune signatures.

Interestingly, the tumors from different organs (colon tumors, 30C vs. endometrial tumors, 30E) of patient #30 had different tumor immune signatures (30C immune excluded vs. 30E immune desert, **Figure 1B**). KEGG pathway enrichment of upregulated genes and downregulated genes showed distinct signaling pathways in the two tumors (**Figures 4A–D**). The deconvolution analysis of RNA-seq data revealed that the two tumors had infiltration of different immune cell types (**Figure 2B**). Moreover, detection of the 12-chemokine signature of TLSs illustrated that the chemokines in the two tumors were totally different (**Figure 3A**). Together, these results support that the tumors from different organs of the same patient may have unique tumor immune signatures, which may be due to the various TMEs in different organs. This effect should be taken into account in ICT in the future.

### The tumor somatic mutations contributed to the variation of the tumor immune signatures in Lynch syndrome

Somatic mutations are defined as mutations specifically found in tumors but not in the surrounding normal tissues (36). We sought to elucidate the effect of somatic mutations *via* WES and relate these data to corresponding tumor immune signatures. The somatic mutations within exon regions showed radical differences in numbers of mutation (**Table S3**) and the proportions of Indel (insertion and deletion) and SNV (single-nucleotide variation).

To decipher the influence of somatic mutations within exons, the functional enrichments of the mutation genes were performed. Mutations in a particular pathway usually means impairment in such a pathway. Interestingly, the signal pathways found in somatic mutations (**Figure 4E**, top) were similar to the downregulated signaling identified in transcriptome of the same specimen (**Figure 4E**, middle), indicating that the dysfunction of signaling is attributed to the somatic mutation. Collectively, the pathways of somatic mutation in the different colon tumors represented distinct features, leading to the variation of the tumor immune signatures (**Figure 4E**). In conclusion, somatic mutations leading to deficiency of certain signaling pathways drive tumor development toward an opposite subtype.

### The activity of immune responses was important for the development of colorectal cancer and patient survival

**Figure 5:**
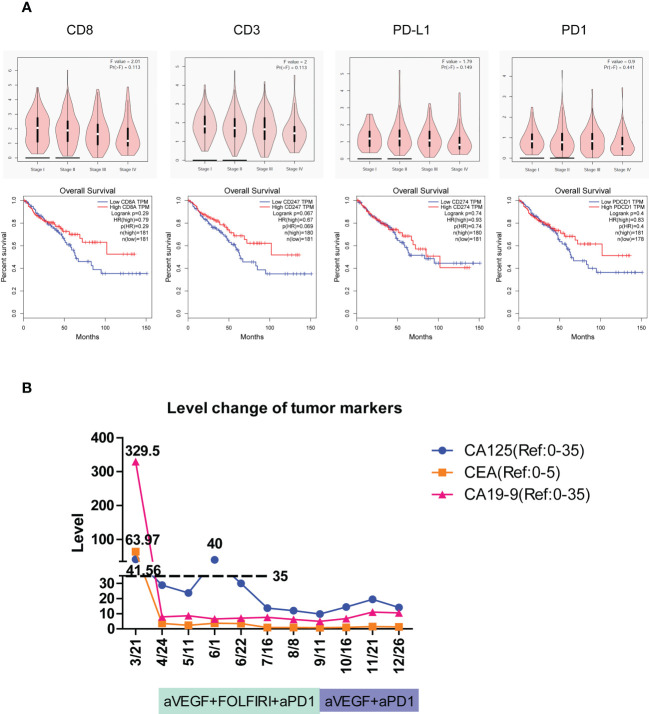
The tumor immune signatures may be potentially applied to predict the development of colorectal cancer, patient survival, and the responsiveness of immune checkpoint inhibition. **(A)** According to GEPIA of TCGA data, the expression of immune response genes including CD8, CD3, PD-L1, and PD-1 is gradually reduced with the development of colorectal cancer from stage I through stage IV. The overall survival of patients with colorectal cancer is elevated in high-expression groups of CD8, CD3, PD-L1, and PD-1 compared to that in low-expression groups. HR, hazard ratio. **(B)** The level change of three cancer markers during combination therapy of anti-PD-1 on patient #2C of immune excluded indicates the complete response.

To detect the effect of immune activity in colorectal cancer, the relationships between immune genes’ level, cancer progression, and patient survival were determined. According to GEPIA (37) of TCGA data, the expression of immune response genes including CD8, CD3, PD-L1, and PD-1 all seemed to have a downward trend with the development of colorectal cancer from stage I to stage IV, although no significance was found (**Figure 5A**, top). Furthermore, the overall survival of patients with colorectal cancer had a relatively higher percentage in high-expression groups of CD8, CD3, PD-L1, and PD-1 than that in low-expression groups (**Figure 5A**, bottom). Collectively, the activity of immune responses indicated by the expression of CD3, CD8, PD-L1, and PD-1 genes was important in the prediction of the progression of colorectal cancer and patient survival.

### LS patients benefited from the prediction and therapy of immune checkpoint inhibition

The colon tumor in patient #2C was at the stage of T4bN1a and had peritoneal metastases. Molecular analysis revealed that the patient carried the PIK3CA H1047R cancer mutation and the POLE F699fs*11 mutation that is associated with high TMB and may benefit from immune checkpoint inhibition (38, 39). The patient had a TMB of 67 mutations/Mb, which is much higher than the medium TMB level of 4.5 mutations/Mb in colon cancer (40). On March 21, 1 month after the tumors were surgically removed, the imaging assessment showed PD in patient #2C. The mRNA profile of predicting clinical response to PD-1 blockade (32, 33) showed the strongest immune activation in inflamed tumors, a medium immune response in immune excluded tumors, and the weakest immune response in immune desert tumors. Patient #2C was identified as immune excluded in tumor immune signatures and may effectively respond to therapy of PD-1 blockade. According to the Keynote177 study, since April 24, patient #2C has received six cycles of combination therapy of anti-VEGF, FOLFIRI, and anti-PD-1, and then four cycles of combination therapy of anti-VEGF and anti-PD-1 (**Figure 5B**). Evaluation of the three tumor markers CA125, CEA, and CA19-9 suggested complete response (CR) after combination therapy (**Figure 5B**). To date, no cancer recurrence has been detected in the patient more than 1 year after the termination of treatment. The data indicated that typing the tumor immune signatures in LS is valuable in the prediction of ICI response and can potentially be applied in the clinic.

## Discussion

Through histology and/or signal feature analysis, the tumors of MMR deficiency in two different cohorts both matched the categorization of the tumor immune signatures: inflamed tumor, immune excluded tumor, and immune desert tumor. Furthermore, the categorization of the tumor immune signatures corresponded to unique infiltrating lymphocyte populations. Strikingly, the tumor immune signature had their specificities in both individuals and organs, suggesting the necessity of the tumor immune signature classification of MMR-deficient tumors before immunotherapy. TLSs have been reported to be positively correlated with better immunotherapy responses and improved patient survival in multiple solid tumors (26–29) and are critical for ICI response prediction. Various tumor immune signatures showed the distinct status of TLSs through the analysis of the 12-chemokine signature (31) in transcriptome. In line with the status of TLSs in immune checkpoint inhibition (30), poorly responding immune desert tumors are associated with immature TLSs. Together, these results indicate that the tumor immune signatures are the same as TLSs in predicting the immunotherapy responses.

Though the exceptionally high burden of somatic mutations in LS was favored in the anti-PD-1 treatment of patients across 12 different tumor types including colorectal cancer (41, 42), approximately half of the patients with MMR defect did not respond to ICT; only 53% of MMR-deficient patients showed objective radiographic responses, and 21% of patients had CRs (42). The findings in this study suggest that TMB and MMR defect are all independent immune signatures, and their sensitivity as reliable indicators of immunotherapy in MMR-deficient tumors needs to be further improved. The immune signature typing by bulk RNA-seq or T-cell staining can easily distinguish the tumor responsiveness to immunotherapy in MMR-deficient tumors, comparable to the TLSs. Furthermore, the immune signature seems to be more sensitive than previously published transcriptional signatures—T cell-inflamed GEPs—in identifying responders to ICT. The analysis of immunohistology of 82 patients with MMR defect in this study revealed that 67.1% (27/82 of inflamed pattern and 28/82 of immune excluded) of the patients may be responsive to the blockade of immune checkpoint in cancer therapy. The tumor immune signatures represent one easy and robust tool in immunotherapy prediction.

A novel finding in this study is that functional enrichment of somatic mutation displayed opposite features of mRNA expression, indicating that the personalized tumor immune signatures resulted from the different features of intratumorally somatic mutation (especially Indel). For instance, for patient 17C, which is of the immune desert type, we found that genes active in the immune excluded or inflamed were mutated. While MMR deficiency greatly increased genomic mutation rate, the really oncogenic process is dependent on somatic mutations, which define tumorigenic properties, the oncogenic program, and the host immune response. The activity of immune responses indicated by the expression of CD3, CD8, PD-L1, and PD-1 genes, which have similar distribution in tumors, exhibited strong correlation with the development of colorectal cancer and patient survival. Our data also reveal that patients with immune excluded tumor can have a good response to the combination therapy of immune checkpoint inhibition. Importantly, the tumor immune signatures in MMR-deficient tumors are valuable to predict the effect of anti-PD-1/PD-L1 therapy (1). Owing to the limited number of patients, the findings in this study need to be further validated in the future.

We have shown that MMR-deficient tumors exhibited personalized tumor immune signatures, which were derived from intratumor somatic mutations. The category of tumor immune signatures based on histology and signal features likely represents a robust and novel strategy to distinguish the subset of patients with MMR deficiency who may benefit from later immune checkpoint inhibition.

## Data availability statement

The datasets presented in this study can be found in online repositories. The names of the repository/repositories and accession number(s) can be found in the article/**Supplementary Material**.

## Ethics statement

The studies involving human participants were reviewed and approved by the Medical Ethics Committee of the Seventh Affiliated Hospital of Sun Yat-sen University. The patients/participants provided their written informed consent to participate in this study. Written informed consent was obtained from the individual(s) for the publication of any potentially identifiable images or data included in this article.

## Author contributions

GZ and CMZ jointly directed this work. Acquisition, analysis, or interpretation of data: All authors. Drafting of the manuscript: GZ and CMZ. Statistical analysis: YSL, QZ, and GZ. All authors contributed to the article and approved the submitted version.

## Supplementary material

Supplementary Figure 1 The immunostaining reveals various immune response patterns in different patients or different organs. The histology of MHC I, PD-1, and its ligand PD-L1 exhibited particular distribution in various patients or organs, same as the analysis of the tumor immune signatures in the mRNA profile. Magnification: 50×.
