# Supplementary figures and images for: Immune desert in MMR-deficient tumors predicts poor responsiveness of immune checkpoint inhibition
Source: Front Immunol. 2023 Apr 28;14:1142862. doi: 10.3389/fimmu.2023.1142862 (PMC10175608; doi:10.3389/fimmu.2023.1142862)

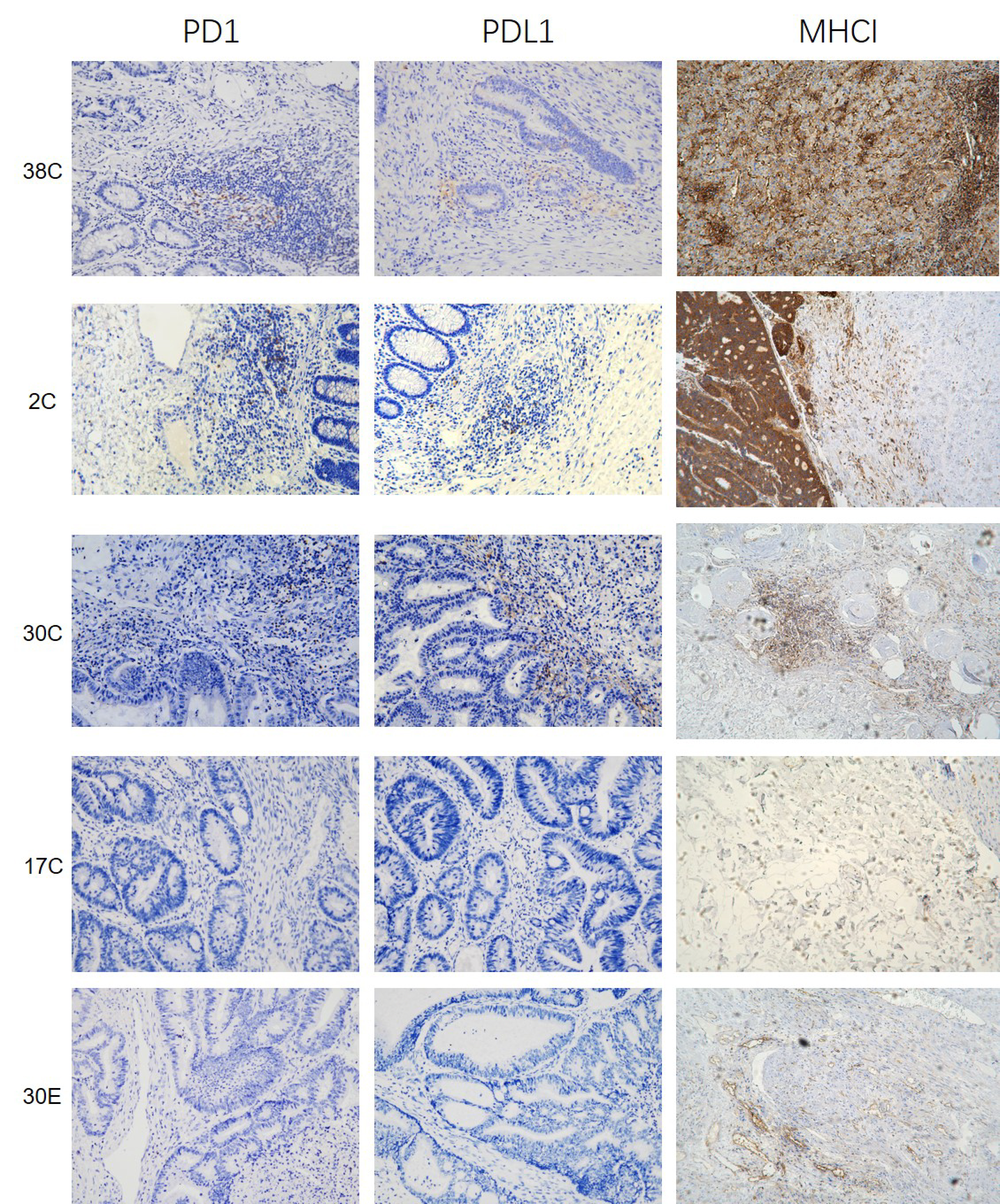

Supplement: Supplementary Figure 1 — The immunostaining reveals various immune response patterns in different patients or different organs. The histology of MHC I, PD-1, and its ligand PD-L1 exhibited particular distribution in various patients or organs, same as the analysis of the tumor immune signatures in the mRNA profile. Magnification: 50×. [file Image_1.jpeg]
