# Supplementary material for: Immune desert in MMR-deficient tumors predicts poor responsiveness of immune checkpoint inhibition
Source: Front Immunol. 2023 Apr 28;14:1142862. doi: 10.3389/fimmu.2023.1142862 (PMC10175608; doi:10.3389/fimmu.2023.1142862)
Supplement: Supplementary file 2 [file Table_1.docx]

**Table S1.** The tumor immune signatures in 82 patients of MMR deficiency

| **IS** | **#** | **Age** | **Sex** | **MMR defects** | **MSI** | **Metastasis** | **Size（cm）** | **Location** | **Type** | **Stage** | **Infiltration** |
| --- | --- | --- | --- | --- | --- | --- | --- | --- | --- | --- | --- |
| ID | #1 | 26 | M | MSH2, MSH6 | UD |  | 0.3 | TC | DA |  |  |
| ID | #2 | 38 | F | MSH2, MSH6 | UD |  | 1 | Hepatic flexure of colon | DA |  |  |
| ID | #5 | 66 | M | MLH1, PMS2, MSH6, MSH2 | UD | unseen |  | SC | LDA |  | SL |
| ID | #7 | 36 | M | MLH1, PMS2 | UD | LNM（6/8） | 1.5;7x4.5 | IP colon | MLDA |  | DM；RSLAT |
| ID | #8 | 31 | F | MSH2, MSH6 | UD | unseen | 4 | DC | MLDA |  | WLIW |
| ID | #9 | 38 | M | MLH1, PMS2 | UD | unseen | 6x4 | colon | MLDA |  | WLIW, RAT，LL7:L51 |
| ID | #10 | 65 | F | MLH1, PMS2 | UD | unseen | 14X9X3.5 | colon | MA, MD |  | WLIW, Not BTSL |
| ID | #11 | 50 | M | MLH1, PMS2 | UD | all LNM | 3 | jejunum | MDA |  | WLIW |
| ID | #12 | 70 | F | MLH1, PMS2 | UD | fibroadpocyte tissue metastasis | 7x5x5 | fibroadipocyte tissue | A |  |  |
| ID | #14 | 68 | M | MLH1, PMS2 | UD | unseen | 14 | colon | DA |  | WLIW |
| ID | #15 | 71 | M | MLH1, PMS2 | UD | unseen | 12x10x6 | AC | MLDA |  | WLIW, RAT |
| ID | #22 | 78 | F | MLH1, PMS2 | UD | unseen |  | IP | MA, MD |  | DM |
| ID | #24 | 58 | F | MLH1, PMS2 | UD | liver S4，S6 metastasis, LNM (2/11) |  | RH | MDA |  | WLIW, RAT |
| ID | #28 | 57 | M | MLH1, PMS2 | UD | unseen |  | AC | A |  | WLIW |
| ID | #30 | 39 | M | MLH1, PMS2 | UD | LNM（1/7） |  | colon | MA |  | WLIW, RAT |
| ID | #43 | 32 | M | MLH1, PMS2 | UD | all LNM（3/3） |  | IP colon | LDA | pT3N2 | WLIW |
| ID | #45 | 51 | M | MSH2 | UD | LNM（1/9） |  | RH | MDA | pT4 | SL |
| **IS** | **#** | **Age** | **Sex** | **MMR defects** | **MSI** | **Metastasis** | **Size（cm）** | **Location** | **Type** | **Stage** | **Infiltration** |
| ID | #47 | 56 | M | MLH1, PMS2 | UD | metastatic to lung, skin, stomach, spleen |  | colon | MLDA |  | WLIW |
| ID | #53 | 33 | M | MLH1, PMS2, MSH6 | UD | LNM（1/13） |  | cecum | MA, MD |  | WLIW, BTSL |
| ID | #56 | 56 | F | MLH1, PMS2 | UD | unseen |  | colon | MDA | PT3N0 | WLIW |
| ID | #62 | 40 | M | MSH2, MSH6 | UD | LNM（1/14） |  | RH | MDA | T3 | WLIW, RSLAT, Not BTSL, |
| ID | #63 | 46 | F | MLH1, PMS2 | UD | LNM（1/6） |  | IP | MLDA |  | WLIW, BTSL |
| ID | #69 | 37 | M | MSH2, MSH6 | UD | LNM |  |  | LDA | T4a | WLIW, BTSL |
| ID | #70 | 56 | F | MLH1, PMS2 | MSI-H | LNM（2/9） |  | colon | MA | pT3N1 | RSLAT |
| ID | #74 | 55 | M | MLH1, PMS2 | UD | LNM（1/16） |  | cecum | MDA |  | WLIW, BTSL |
| ID | #75 | 61 | M | MLH1, PMS2 | UD | LNM（1/7） |  | colon | MLDA |  | SL |
| ID | #77 | 29 | M | MSH2, MSH6 | UD | LNM（1/3） |  | SC | MDA |  |  |
| IE | #4 | 38 | M | MSH2, MSH6 | UD | unseen |  | colon | DA |  | DM |
| IE | #6 | 71 | F | MLH1, PMS2 | UD | unseen |  | TC | LDA |  | WLIW, RAT |
| IE | #13 | 68 | M | MLH1, PMS2 | UD | unseen | 6x5x1.2 | colon | MLDA |  | WLIW, BTSL |
| IE | #16 | 64 | F | MLH1, PMS2, MSH6, MSH2 | UD | unseen |  | colon | MHDA |  | WLIW |
| IE | #20 | 48 | M | MSH2, MSH6 | MSI-H | unseen |  | rectum | MDA |  | WLIW, RAT |
| IE | #23 | 55 | F | MSH2, MSH6 | MSI-H | unseen |  | IP | MDA |  | WLIW, Not BTSL |
| IE | #25 | 62 | F | MLH1, PMS2 | MSI-H | unseen |  | RH | MDA |  | RSL, Not BTSL |
| IE | #26 | 56 | M | MLH1, PMS2 | UD | unseen |  | Colon; rectum | MDA，HDA |  | adipose tissue |
| **IS** | **#** | **Age** | **Sex** | **MMR defects** | **MSI** | **Metastasis** | **Size（cm）** | **Location** | **Type** | **Stage** | **Infiltration** |
| IE | #29 | 56 | M | MLH1 | UD | unseen |  | SC | MDA |  | RAT |
| IE | #31 | 44 | M | MLH1, PMS2 | UD | unseen |  | colon | MDA |  | WLIW, RAT |
| IE | #34 | 42 | M | MLH1, PMS2 | UD | unseen |  | SC | MDA |  | WLIW, RSLAT |
| IE | #35 | 26 | M | MLH1, PMS2 | UD | LNM（1/4） |  | AC | MDA |  | WLIW, RSLAT |
| IE | #42 | 74 | F | PMS2 | UD | unseen |  | cecum | MDA |  | WLIW, Not BTSL |
| IE | #46 | 65 | M | PMS2 | MSI-H | unseen |  | RH | MLDA | pT3N0 | SL |
| IE | #48 | 40 | M | MSH6 | UD | unseen |  | AC | MDA | pT3NO | WLIW, RAT |
| IE | #50 | 59 | M | MSH2, MSH6 | UD | unseen |  | small intestine - colon anastomotic | MDA |  | WLIW, Not BTSL |
| IE | #52 | 30 | M | MSH2, MSH6 | UD | unseen |  | colon | MDA | PT4a | WLIW, BTSL |
| IE | #55 | 58 | M | MLH1, PMS2 | UD | LNM（3/9） |  | RH | MLDA | T3 | DM, partially RAT, not RSL |
| IE | #59 | 67 | F | MLH1, PMS2 | UD | unseen |  |  | MDA |  | BTSL |
| IE | #60 | 56 | M | MLH1, PMS2 | UD | unseen |  | DC | MDA | pT2N0 | muscularis |
| IE | #61 | 68 | F | MLH1, PMS2 | Lynch | LNM（1/5） |  | colon | LDA |  | WLIW, lynch |
| IE | #64 | 37 | M | MSH6 | UD | unseen |  | rectum | MDA |  | muscularis |
| IE | #66 | 56 | F | PMS2 | UD | unseen |  | colon | MDA | T3 | WLIW, RSL, not BTSL |
| IE | #68 | 57 | M | MSH2 | UD | unseen |  | SBJ | MDA |  | WLIW, BTSL |
| **IS** | **#** | **Age** | **Sex** | **MMR defects** | **MSI** | **Metastasis** | **Size（cm）** | **Location** | **Type** | **Stage** | **Infiltration** |
| IE | #71 | 55 | F | MLH1, PMS2 | UD | unseen |  | colon | LDA | pT4aN0 | WLIW, BTSL |
| IE | #73 | 76 | M | MLH1, PMS2 | UD | unseen |  | rectum | MLDA | pT2 | reach DM |
| IE | #79 | 54 | F | MSH2, MSH6 | UD | unseen |  | SBJ | MDA |  | WLIW |
| IE | #80 | 47 | M | MLH1, PMS2 | UD | unseen |  | AC | LDA |  | WLIW, BTSL |
| I | #3 | 27 | M | MSH2 | UD |  |  | DC | A |  |  |
| I | #17 | 49 | M | MLH1, PMS2 | MSI-H | LNM（1/14） |  | RH | MLDA |  | WLIW, adipose tissue |
| I | #18 | 66 | M | MLH1, PMS2 | UD |  |  | colon | MLDA |  | WLIW, RAT |
| I | #19 | 59 | M | PMS2 | UD | unseen |  | colon | MLDA |  | WLIW, RSLAT |
| I | #21 | 44 | M | PMS2 | MSI-H | unseen |  | AC | MDA |  | RSL, Not BTSL |
| I | #27 | 38 | F | MLH1, PMS2 | UD | unseen |  | colon | MLDA |  | WLIW, RAT |
| I | #32 | 54 | F | MSH2, MSH6 | MSI-H | unseen |  | colon | MA |  | muscularis |
| I | #33 | 42 | M | PMS2 | UD | unseen |  | colon | MDA |  | DM |
| I | #36 | 72 | F | MLH1, PMS2 | UD | unseen |  | RH | LDA |  | SL firbosis tissues |
| I | #37 | 54 | F | MLH1, PMS2 | UD | unseen |  | DC- SC | MDA |  | WLIW, Not BTSL |
| I | #38 | 67 | M | MSH2, MSH6 | UD | unseen |  | AC | MDA |  | DM |
| I | #39 | 40 | M | MSH6 | UD | unseen |  | colon | MLDA |  | SL |
| I | #40 | 37 | M | MSH2, MSH6 | UD | unseen |  | colon | MDA |  | WLIW, Not BTSL |
| I | #41 | 58 | M | MLH1, PMS2 | UD | unseen |  | Splenic flexure of colon | LDA |  | WLIW, not RAT |
| **IS** | **#** | **Age** | **Sex** | **MMR defects** | **MSI** | **Metastasis** | **Size（cm）** | **Location** | **Type** | **Stage** | **Infiltration** |
| I | #44 | 52 | M | MLH1, PMS2 | UD | unseen |  | Sigmoid colon | MDA |  | WLIW, BTSL |
| I | #49 | 77 | F | MSH2, MSH6 | UD | unseen |  | rectum | MLDA |  | WLIW |
| I | #51 | 78 | F | MLH1, PMS2 | MSI-H | unseen |  | RH | LDA |  | WLIW, BTSL |
| I | #54 | 55 | M | MLH1, PMS2 | UD | unseen |  | DC | MA | pT3N0 | WLIW, Not BTSL |
| I | #57 | 70 | M | MLH1, PMS2 | UD | unseen |  |  | MLDA | PT3N0 | RSL |
| I | #58 | 38 | M | MLH1, PMS2 | UD | unseen |  | jejunum | LDA | pT4a | WLIW, BTSL |
| I | #65 | 30 | M | MSH2 | UD | unseen |  | LH | MDA | pT3N0 | RSLAT |
| I | #67 | 45 | M | MLH1, PMS2 | UD | multiple LNM（4/6） |  | colon | MLDA | T4aN2b | WLIW, SLAT |
| I | #72 | 26 | M | MLH1, PMS2 | UD | unseen |  | RH | MHDA |  | WLIW, RSLAT |
| I | #76 | 44 | M | MLH1, PMS2 | UD | unseen |  | SC | MDA |  | muscularis |
| I | #78 | 43 | M | PMS2 | UD | unseen |  | cecum | MLDA | pT2 | DM |
| I | #81 | 64 | F | MSH2 | UD | LNM（1/4） |  | cecum | MDA | pT4 | cecum, SL |
| I | #82 | 33 | M | MLH1, PMS2 | UD | unseen |  | colon | MDA | pT4aN0 | RSLAT |

Abbreviations: IS, tumor immune signatures; ID, immune desert; IE, immune excluded, I, inflamed; M, male; F, Female; UD, undetected; LNM, lymph nodes metastatics; RH, Right hemi-colon; LH, Left hemi-colon; IP, Ileocecal part; SBJ, Straight B junction; TC, Transverse colon; AC, Ascending colon; DC, Descending colon; SC, Sigmoid colon; A, adenocarcinoma; DA, differentiated adenocarcinoma; MA, Mucinous adenocarcinoma; MD, Middle differentiated; LDA, Low differentiated adenocarcinoma; MDA, Middle differentiated adenocarcinoma; HDA, high differentiated adenocarcinoma; MLDA, Middle low differentiated adenocarcinoma; MHDA, Middle high differentiated adenocarcinoma; WLIW, Whole layer of intestinal wall; BTSL, break through the serous layer; RAT, reach adipose tissue; RSLAT, reach the serous layer adipose tissue; SL, the serous layer; RSL, reach the serous layer; DM, Deep muscularis.

**Table S2.** Clinical characteristics of Lynch syndrome (LS) patients

| **Patient#** | **Sex** | **Age** | **IHC lost** | **MSI** | **Germline mutation** | **Family history** | **Tumors** |
| --- | --- | --- | --- | --- | --- | --- | --- |
| **17C** | M | 31 | MLH1, PMS2 | MSI-H | MLH1 Het mutation: NM_000249. c.1273dupA (p.R425fs*5) | yes | colon |
| **30C/30E** | F | 64 | MSH2 | MSI-H | MSH2 Het deletion of exon11-16 | yes | colon, endometrium, ureter |
| **38C** | M | 41 | MLH1, PMS2 | MSI-H | MLH1 Het mutation: NM_000249. c.588delA (p.K196fs*6) | unknown | colon |
| **2C** | F | 26 | MLH1, PMS2 | MSI-H | MLH1 Het mutation: NM_000249. c.2133delC (p.W712fs) | unknown | colon |
| **8C** | M | 30 | PMS2 | NA | NA | unknown | colon |
| **41C** | M | 53 | MSH2 | NA | NA | unknown | colon |
| **CC0518** | M | 54 | MLH1, PMS2 | NA | NA | unknown | colon |
| **CC0527** | M | 46 | MSH6 | NA | NA | unknown | colon |
| **CC0429** | F | 64 | MSH2 | MSI-H | NA | unknown | colon |
| **CC124** | M | 34 | MSH2，MSH6 | MSI-H | NA | unknown | colon |

Abbreviations: M, male; F, female; NA, not assessed;

**Table S3.** The statistics of somatic mutations

| **Sample Name** | **Exon mutations** | **Indel** | **SNV** |
| --- | --- | --- | --- |
| 17C | 967 | 194 (20.1%) | 773 (79.9%) |
| 38C | 194 | 16 (8.2%) | 178 (91.8%) |
| 30C | 4230 | 694 (16.4) | 3536 (83.6) |
| 30E | 745 | 84 (11.3%) | 661 (88.7%) |
| 2C | 1453 | 232 (16.0) | 1221 (84.0%) |

Abbreviations: Indel, insertion and deletion; SNV, single nucleotide variation.

**Table S4.** The potential benefit of prior testing of response to immunotherapy

| **Cancer Type** | **Therapy** | **Index** | **Total (n_R/T_ %)** | **Assess (n_R/T_ %)** | | | **Reference** |
| --- | --- | --- | --- | --- | --- | --- | --- |
| metastatic melanoma | PD-1 blockade | CD8 high | 9/15 60% | 9/11 81.8% | | | Paul C. Tumeh et al. 2014 Nature |
| NSCLC | PD-L1 blockade | PD-L1 high | 12/53 23% | 6/13 46.2% | | | Roy S. Herbst et al. 2014 Nature |
| all tumors | PD-L1 blockade | PD-L1 high | 36/175 21% | 19/56 33.9% | | | Roy S. Herbst et al. 2014 Nature |
| metastatic urothelial cancer | PD-L1 blockade | Immune type | 61/244 25% | Inflamed | Excluded | Desert | Sanjeev Mariathasan et al. 2018 Nature |
|  |  |  |  | 19/62 30.6% | 28/113 24.8% | 14/69 20.3% |  |

NSCLS: non-small cell lung cancer n: number R: response T: total patient number
