# Supplementary material for: Immune desert in MMR-deficient tumors predicts poor responsiveness of immune checkpoint inhibition
Source: Front Immunol. 2023 Apr 28;14:1142862. doi: 10.3389/fimmu.2023.1142862 (PMC10175608; doi:10.3389/fimmu.2023.1142862)
Supplement: Supplementary file 3 [file Table_2.docx]

TCGA_READ_TUMOR：

| TCGA_DC_6683_01 | TCGA_AF_6672_01 | TCGA_AF_2690_01 | TCGA_EI_7004_01 | TCGA_F5_6464_01 | TCGA_EI_6509_01 | TCGA_AG_3591_01 | TCGA_DT_5265_01 | TCGA_DC_6682_01 | TCGA_DY_A1DF_01 | TCGA_DC_6154_01 | TCGA_EI_7002_01 | TCGA_AF_2687_01 | TCGA_AF_6136_01 | TCGA_EI_6512_01 | TCGA_DC_6681_01 | TCGA_AF_A56L_01 | TCGA_AF_6655_01 | TCGA_EI_6514_01 | TCGA_EF_5831_01 | TCGA_AH_6547_01 | TCGA_F5_6702_01 | TCGA_DC_6155_01 | TCGA_DY_A1H8_01 | TCGA_AF_5654_01 | TCGA_DC_6160_01 | TCGA_CL_4957_01 | TCGA_EI_6507_01 | TCGA_AG_3742_01 | TCGA_DC_5869_01 | TCGA_EI_6884_01 | TCGA_DC_6156_01 | TCGA_DY_A1DC_01 | TCGA_CI_6622_01 | TCGA_EI_6882_01 | TCGA_F5_6811_01 | TCGA_AH_6897_01 | TCGA_F5_6813_01 | TCGA_F5_6812_01 | TCGA_AG_3592_01 | TCGA_AF_3911_01 | TCGA_EI_6508_01 | TCGA_DC_6158_01 | TCGA_DY_A1DD_01 | TCGA_CL_5917_01 | TCGA_EI_6506_01 | TCGA_F5_6814_01 | TCGA_CI_6620_01 | TCGA_EI_6513_01 | TCGA_F5_6864_01 | TCGA_AH_6903_01 | TCGA_AH_6643_01 | TCGA_F5_6465_01 | TCGA_G5_6233_01 | TCGA_DC_6157_01 | TCGA_DY_A0XA_01 | TCGA_DC_4749_01 | TCGA_G5_6572_02 | TCGA_DC_5337_01 | TCGA_G5_6235_01 | TCGA_EI_6917_01 | TCGA_EF_5830_01 | TCGA_CI_6623_01 | TCGA_AH_6549_01 | TCGA_EI_6510_01 | TCGA_AH_6544_01 | TCGA_AG_4021_01 | TCGA_EI_6511_01 | TCGA_CI_6621_01 | TCGA_BM_6198_01 | TCGA_F5_6863_01 | TCGA_F5_6861_01 | TCGA_AG_3732_01 | TCGA_AF_A56N_01 | TCGA_CL_5918_01 | TCGA_F5_6571_01 | TCGA_AF_4110_01 | TCGA_G5_6572_01 | TCGA_AG_3731_01 | TCGA_AG_4022_01 | TCGA_AH_6644_01 | TCGA_DY_A1DG_01 | TCGA_EI_6885_01 | TCGA_EI_6881_01 | TCGA_AG_3725_01 | TCGA_CI_6624_01 | TCGA_CI_6619_01 | TCGA_DY_A1DE_01 | TCGA_DC_4745_01 | TCGA_AF_A56K_01 | TCGA_G5_6641_01 | TCGA_AF_2693_01 |

TCGA_READ_TUMOR：

| TCGA_AD_5900_01 | TCGA_D5_5540_01 | TCGA_CK_4947_01 | TCGA_F4_6855_01 | TCGA_CM_6164_01 | TCGA_A6_A565_01 | TCGA_DM_A28F_01 | TCGA_AZ_6601_01 | TCGA_CM_5868_01 | TCGA_RU_A8FL_01 | TCGA_G4_6298_01 | TCGA_AA_3660_01 | TCGA_D5_6928_01 | TCGA_DM_A1HB_01 | TCGA_NH_A5IV_01 | TCGA_5M_AAT4_01 | TCGA_AD_6888_01 | TCGA_D5_6533_01 | TCGA_CM_6170_01 | TCGA_CM_6172_01 | TCGA_G4_6299_01 | TCGA_CK_5912_01 | TCGA_4T_AA8H_01 | TCGA_AZ_4315_01 | TCGA_G4_6625_01 | TCGA_QG_A5YX_01 | TCGA_A6_5664_01 | TCGA_AA_3663_01 | TCGA_G4_6588_01 | TCGA_A6_2685_01 | TCGA_DM_A1D9_01 | TCGA_D5_6539_01 | TCGA_CM_6675_01 | TCGA_AA_3492_01 | TCGA_AZ_6607_01 | TCGA_D5_6541_01 | TCGA_F4_6807_01 | TCGA_CM_6161_01 | TCGA_A6_5657_01 | TCGA_AZ_6608_01 | TCGA_NH_A50U_01 | TCGA_CM_4751_01 | TCGA_CM_5349_01 | TCGA_AA_3509_01 | TCGA_CM_6171_01 | TCGA_NH_A8F8_01 | TCGA_AA_3675_01 | TCGA_CK_5915_01 | TCGA_DM_A1HA_01 | TCGA_NH_A8F7_01 | TCGA_D5_6538_01 | TCGA_F4_6570_01 | TCGA_QG_A5Z2_01 | TCGA_G4_6627_01 | TCGA_AD_6548_01 | TCGA_A6_6653_01 | TCGA_D5_6537_01 | TCGA_CK_6751_01 | TCGA_AY_A69D_01 | TCGA_CM_6168_01 | TCGA_G4_6304_01 | TCGA_DM_A1DB_01 | TCGA_CK_6747_01 | TCGA_D5_6930_01 | TCGA_AA_3662_01 | TCGA_A6_6140_01 | TCGA_DM_A1DA_01 | TCGA_G4_6311_01 | TCGA_WS_AB45_01 | TCGA_NH_A8F7_06 | TCGA_D5_6536_01 | TCGA_G4_6315_01 | TCGA_NH_A50V_01 | TCGA_D5_6924_01 | TCGA_G4_6309_01 | TCGA_A6_6138_01 | TCGA_D5_6540_01 | TCGA_F4_6856_01 | TCGA_4N_A93T_01 | TCGA_CA_6719_01 | TCGA_D5_6931_01 | TCGA_AU_6004_01 | TCGA_DM_A28E_01 | TCGA_CA_5797_01 | TCGA_CM_4744_01 | TCGA_G4_6321_01 | TCGA_AA_A01X_01 | TCGA_AY_6197_01 | TCGA_AZ_4615_01 | TCGA_AA_3496_01 | TCGA_D5_6920_01 | TCGA_CA_6718_01 | TCGA_CM_5348_01 | TCGA_CK_4950_01 | TCGA_AY_6196_01 | TCGA_AZ_4614_01 | TCGA_AD_6963_01 | TCGA_F4_6461_01 | TCGA_CM_5861_01 | TCGA_D5_6530_01 | TCGA_F4_6805_01 | TCGA_D5_5537_01 | TCGA_NH_A50T_01 | TCGA_F4_6854_01 | TCGA_DM_A28A_01 | TCGA_D5_5541_01 | TCGA_G4_6626_01 | TCGA_CM_5344_01 | TCGA_A6_6782_01 | TCGA_CM_5864_01 | TCGA_D5_6529_01 | TCGA_AZ_5407_01 | TCGA_DM_A28C_01 | TCGA_QG_A5Z1_01 | TCGA_G4_6306_01 | TCGA_CA_5256_01 | TCGA_CM_6678_01 | TCGA_AA_3685_01 | TCGA_CM_6169_01 | TCGA_T9_A92H_01 | TCGA_AA_3502_01 | TCGA_G4_6310_01 | TCGA_D5_5538_01 | TCGA_CM_5862_01 | TCGA_F4_6809_01 | TCGA_AZ_4616_01 | TCGA_AZ_4313_01 | TCGA_A6_A566_01 | TCGA_AA_3511_01 | TCGA_D5_7000_01 | TCGA_AZ_5403_01 | TCGA_D5_6923_01 | TCGA_AZ_6605_01 | TCGA_CK_4951_01 | TCGA_AZ_6606_01 | TCGA_G4_6295_01 | TCGA_A6_6137_01 | TCGA_DM_A1D7_01 | TCGA_CM_6163_01 | TCGA_G4_6586_01 | TCGA_AA_3489_01 | TCGA_D5_6922_01 | TCGA_DM_A1D8_01 | TCGA_F4_6806_01 | TCGA_CM_6676_01 | TCGA_AA_3506_01 | TCGA_CM_6166_01 | TCGA_CM_6162_01 | TCGA_DM_A280_01 | TCGA_CK_5916_01 | TCGA_AD_6964_01 | TCGA_AD_6890_01 | TCGA_D5_6898_01 | TCGA_AA_A01Z_01 | TCGA_AZ_4684_01 | TCGA_G4_6628_01 | TCGA_A6_A5ZU_01 | TCGA_AD_6889_01 | TCGA_F4_6460_01 | TCGA_AA_3495_01 | TCGA_D5_6535_01 | TCGA_AA_3712_01 | TCGA_DM_A282_01 | TCGA_AM_5821_01 | TCGA_CM_6165_01 | TCGA_SS_A7HO_01 | TCGA_F4_6569_01 | TCGA_AY_5543_01 | TCGA_DM_A0XD_01 | TCGA_G4_6320_01 | TCGA_G4_6307_01 | TCGA_CK_5913_01 | TCGA_DM_A0XF_01 | TCGA_D5_6932_01 | TCGA_AY_A54L_01 | TCGA_AA_3526_01 | TCGA_D5_6929_01 | TCGA_CM_5863_01 | TCGA_A6_2675_01 | TCGA_F4_6459_01 | TCGA_5M_AAT6_01 | TCGA_AY_6386_01 | TCGA_CA_5255_01 | TCGA_DM_A0X9_01 | TCGA_AA_3655_01 | TCGA_AZ_4682_01 | TCGA_G4_6314_01 | TCGA_A6_A567_01 | TCGA_DM_A1D0_01 | TCGA_A6_2686_01 | TCGA_3L_AA1B_01 | TCGA_AA_A01P_01 | TCGA_G4_6303_01 | TCGA_CA_5796_01 | TCGA_CK_5914_01 | TCGA_DM_A28H_01 | TCGA_F4_6463_01 | TCGA_F4_6703_01 | TCGA_CA_6716_01 | TCGA_QG_A5YV_01 | TCGA_A6_5666_01 | TCGA_G4_6317_02 | TCGA_A6_6654_01 | TCGA_DM_A28K_01 | TCGA_NH_A6GA_01 | TCGA_A6_6651_01 | TCGA_CM_4747_01 | TCGA_AD_6965_01 | TCGA_CM_6677_01 | TCGA_CK_6748_01 | TCGA_DM_A285_01 | TCGA_CM_6674_01 | TCGA_AD_6901_01 | TCGA_AA_A02Y_01 | TCGA_AY_A71X_01 | TCGA_G4_6297_01 | TCGA_CK_4948_01 | TCGA_A6_6142_01 | TCGA_CK_6746_01 | TCGA_G4_6317_01 | TCGA_QL_A97D_01 | TCGA_AZ_6600_01 | TCGA_CM_6167_01 | TCGA_D5_6927_01 | TCGA_NH_A6GC_01 | TCGA_NH_A6GB_01 | TCGA_F4_6808_01 | TCGA_DM_A288_01 | TCGA_D5_6531_01 | TCGA_A6_6652_01 | TCGA_G4_6294_01 | TCGA_AY_A8YK_01 | TCGA_CA_6715_01 | TCGA_AM_5820_01 | TCGA_CA_5254_01 | TCGA_A6_6141_01 | TCGA_CM_6679_01 | TCGA_AZ_6603_01 | TCGA_A6_5667_01 | TCGA_CM_5860_01 | TCGA_A6_5660_01 | TCGA_DM_A28M_01 | TCGA_AZ_4323_01 | TCGA_AA_3697_01 | TCGA_CM_4743_01 | TCGA_F4_6704_01 | TCGA_A6_4105_01 | TCGA_G4_6322_01 | TCGA_DM_A28G_01 | TCGA_AZ_6598_01 | TCGA_G4_6293_01 | TCGA_AD_A5EK_01 | TCGA_AA_A02K_01 | TCGA_AZ_6599_01 | TCGA_D5_6534_01 | TCGA_A6_5662_01 | TCGA_A6_A56B_01 | TCGA_QG_A5YW_01 | TCGA_AD_6895_01 | TCGA_D5_6532_01 | TCGA_D5_6926_01 | TCGA_AU_3779_01 | TCGA_CA_6717_01 | TCGA_AD_6899_01 | TCGA_D5_5539_01 | TCGA_A6_2682_01 | TCGA_DM_A1D4_01 | TCGA_CK_4952_01 | TCGA_5M_AATE_01 | TCGA_G4_6302_01 | TCGA_A6_6648_01 | TCGA_CM_6680_01 | TCGA_A6_6649_01 | TCGA_AD_A5EJ_01 | TCGA_AA_3713_01 |
